# Evolution of progressive multifocal leukoencephalopathy in HIV-infected patients

**DOI:** 10.1186/1471-2334-14-S4-O28

**Published:** 2014-05-29

**Authors:** Ana Maria Iancu, Dalia Sorina Carp, Simona Claudia Cambrea

**Affiliations:** 1 Clinical Hospital of Infectious Diseases, Constanța, Romania; 2 Ovidius University, Constanța, Romania

## 

Progressive multifocal leukoencephalopathy (PML) is a demyelinating disease of the CNS caused by the JC virus. It occurs in immunosuppressed individuals: patients with AIDS, malignancies, autoimmune rheumatological diseases, or those receiving immune therapy with monoclonal antibodies.

Two patients diagnosed at 13 years old with HIV infection, treated with combined active antiretroviral therapy (cART) but with curling adherence in the last years, were diagnosed after 2, respectively 3 years of stopping therapy with PML. Both patients were with a poor immunological status expressed by a low CD4 T-cell count (<50 cells/µL) and high viral load (427,000 copies/mL) at the moment of diagnosis. They presented headache, mild disturbances of fine movements and mild speech disorders without fever, which constantly progressed to hemiparesis, ataxia, dysarthria, aphasia, visual disturbances and swallowing disorders. The cerebrospinal fluid (CSF) was normal, the serology for *Cryptococcus*, Herpes simplex virus, Varicella-Zoster virus, Cytomegalovirus, Epstein Barr virus, *Toxoplasma* and *Treponema pallidum* were negative, the cultures for *Mycobacterium tuberculosis*, bacteria and fungi were negative. MRI scan revealed diffuse lesions in the right cerebellar hemisphere associated with edematous and inflammatory cortico-subcortical lesions located in the right fronto-parietal area with bilateral cerebellar extension and involvement of the pons after one month, in one patient. The other patient presented diffuse lesions located in the fronto-parietal periventricular white matter and corpus callosum. There was no mass effect. On T2-weighted and fluid-attenuated inversion recovery (FLAIR) sequences, the lesions were hyperintense. Polymerase chain reaction (PCR) for JC virus DNA in the CSF was positive for one patient and negative for the other. However, a negative PCR does not exclude this diagnosis. Under cART one patient survived with some neurological disorders, more than 5 years and today is a compliant, undetectable patient; unfortunately the other patient died after 3 months.

For both patients the diagnosis was sustained by the clinical, biological findings and neuroimaging changes on MRI, but only one was confirmed by the JC virus DNA detected in the CSF.

In such cases antiretroviral therapy should be started immediately, in order to achieve a rapid undetectable HIV viral load.

